# The right inferior frontal gyrus processes nested non-local dependencies in music

**DOI:** 10.1038/s41598-018-22144-9

**Published:** 2018-02-28

**Authors:** Vincent K. M. Cheung, Lars Meyer, Angela D. Friederici, Stefan Koelsch

**Affiliations:** 1 Department of Neuropsychology, Max Planck Institute for Human Cognitive and Brain Sciences, Leipzig, Germany; 2 Department of Biological and Medical Psychology, University of Bergen, Bergen, Norway

## Abstract

Complex auditory sequences known as music have often been described as hierarchically structured. This permits the existence of non-local dependencies, which relate elements of a sequence beyond their temporal sequential order. Previous studies in music have reported differential activity in the inferior frontal gyrus (IFG) when comparing regular and irregular chord-transitions based on theories in Western tonal harmony. However, it is unclear if the observed activity reflects the interpretation of hierarchical structure as the effects are confounded by local irregularity. Using functional magnetic resonance imaging (fMRI), we found that violations to non-local dependencies in nested sequences of three-tone musical motifs in musicians elicited increased activity in the right IFG. This is in contrast to similar studies in language which typically report the left IFG in processing grammatical syntax. Effects of increasing auditory working demands are moreover reflected by distributed activity in frontal and parietal regions. Our study therefore demonstrates the role of the right IFG in processing non-local dependencies in music, and suggests that hierarchical processing in different cognitive domains relies on similar mechanisms that are subserved by domain-selective neuronal subpopulations.

## Introduction

Complex auditory sequences known as music exist in all human cultures^1^, and elements in many musical styles are hierarchically structured^2,3^. Examples include harmonic progressions in classical western tonal music^4^ and jazz^5^, as well as transformations of tone rows in twelve-tone serialist compositions^6^. A sequence is said to be hierarchical if the dependencies (rules which bind two elements) between its elements can be represented as a type of mathematical graph called a rooted tree (acyclic graph with a designated root element)^7,8^. This means that all elements of a sequence are connected to form an overarching structure^7^, and implies the existence of a sub-/superordinate relationship between elements^9^. We shall refer to this definition of hierarchy throughout the text. Furthermore, a dependency is said to be *local* if it relates elements that directly follow one another in a sequence, and *non-local* if the dependency spans over multiple intervening elements. Consider nested sequences, which have the form A_n_A_n−1_…A_1_B_1_…B_n−1_B_n_ and contain dependencies that are embedded, or *nested*, within another dependency: In the nested sequence A_3_A_2_A_1_B_1_B_2_B_3_, the (local) dependency between A_1_ and B_1_ is embedded within the (non-local) dependency between A_2_ and B_2_, which is in turn embedded within the (non-local) dependency between A_3_ and B_3_. Importantly, the ability to relate remote, non-local musical events beyond their immediate temporal sequential order is said to be crucial for successfully processing hierarchical structures in music^7,10,11^.

Previous studies on processing hierarchical structures in music argued that humans can differentiate between auditory tone sequences generated according to a hierarchical recursive rule and an iterative rule^12^, show priming effects in integrating harmonic contextual information^13,14^, and discriminate between grammatical and ungrammatical transformations in serialist music^15,16^. Moreover, harmonically irregular chords within a chord sequence were shown to elicit an early right anterior negativity (ERAN) in event-related brain potentials (ERPs) (using EEG^11,17,18^ and MEG for the magnetic equivalent^19^), which can already be observed in infants^20^, and in a musical scale previously unheard by participants^21^. Functional MRI (fMRI) studies employing similar violation paradigms have also reported differential responses in the bilateral inferior frontal gyri (sometimes with a right-hemispheric dominance)^18,22–27^, and the anterior insular cortices^22,23,27^. Activity in the inferior frontal gyrus (IFG) was suggested to process hierarchical structure in music, given that language studies have implicated the left IFG – particularly the pars opercularis – in syntactic reordering and embedding^28–31^. This is corroborated by interference effects observed between musical and linguistic syntax^32–34^, which suggest that processing hierarchical structure relies on domain-general neural resources^35^.

However, the question of whether hierarchical processing was really involved in the previous studies has also been raised. It has been argued that humans rearrange scrambled phrases of music in a way that was grammatically-coherent locally but not globally^36^, and are insensitive towards transpositions^37,38^ and re-orderings^39^ to sections of classical music pieces. Moreover, violations to the hierarchical structure in previous experiments were not restricted to dependencies between non-local elements, but also violated local dependencies between immediately-adjacent chords^11^. Although the ERP study by Koelsch and colleagues^11^ controlled for the possibility of processing musical sequences in a strictly local fashion, the precise neuro-functional basis of processing non-local dependencies in music nevertheless remains unknown.

The current study was thus conducted to assess the functional basis of processing non-local dependencies in music in the human brain, whilst controlling for local transition probabilities. Our approach was a grammaticality judgment task based on an artificial grammar learning paradigm^40–42^. Musicians learnt a nested atonal grammar of piano-tone sequences (i.e. A_n_A_n−1_…A_1_B_1_…B_n−1_B_n_) before discriminating between novel grammatical and ungrammatical musical sequences during fMRI scanning. In contrast to previous studies, successful completion of the task requires participants to explicitly abstract notes into motifs and to store multiple non-local dependencies in parallel. We moreover manipulated the level of embedding (that is, the number of nested dependencies in a sequence) to dissociate the processing of nested dependencies in music from the effects of increasing working memory demands on processing these dependencies, and to ensure that the observed responses in resolving the nested dependencies generalised to different levels.

We hypothesised that violations to the nested grammar would elicit increased BOLD responses in the bilateral inferior frontal gyri, especially in the right hemisphere. Based on the literature on auditory tonal working memory^43–46^, we also hypothesised increased BOLD responses in the dorsolateral prefrontal cortex and parietal areas with increased levels of embedding due to additional working memory demands.

## Materials and Methods

### Participants

Twenty musically-trained participants (12 females, 8 males) with normal hearing and at least seven years of training (cf.^47–49^) in their most experienced instrument (M = 13.30 years, SD = 5.78 years) completed both sessions of the experiment. No participants reported absolute pitch or neurological/psychological disorders. Participants were excluded from further analyses if their hit rates during the fMRI session for grammatical sequences, ungrammatical sequences with category violations, or ungrammatical sequences with state violations (see Stimuli) fell below the 5% significance level of performing above chance according to a binomial test. Two male participants were excluded that way. One female participant was excluded due to incidental findings. Data were analysed for the remaining 17 participants (mean age = 26.29 years, SD = 2.37 years; mean experience in most experienced instrument = 13.76 years, SD = 6.00 years, seven of whom were conservatory-level). They were right-handed^50^, and had a mean score of 93.3 (SD = 13.1) in general music sophistication^51^. Informed consent was obtained from all participants and the experiment was approved by the ethics committee of the University of Leipzig in accordance with the Declaration of Helsinki.

### Stimuli

Our study employed a 2 × 2 factorial design with factors GRAMMATICALITY (GRAMMATICAL versus UNGRAMMATICAL) and LEVEL OF EMBEDDING (LoE: ONE-LoE versus TWO-LoE) to dissociate the effects of grammaticality and working memory in processing non-local dependencies in music.

**Figure 1:**
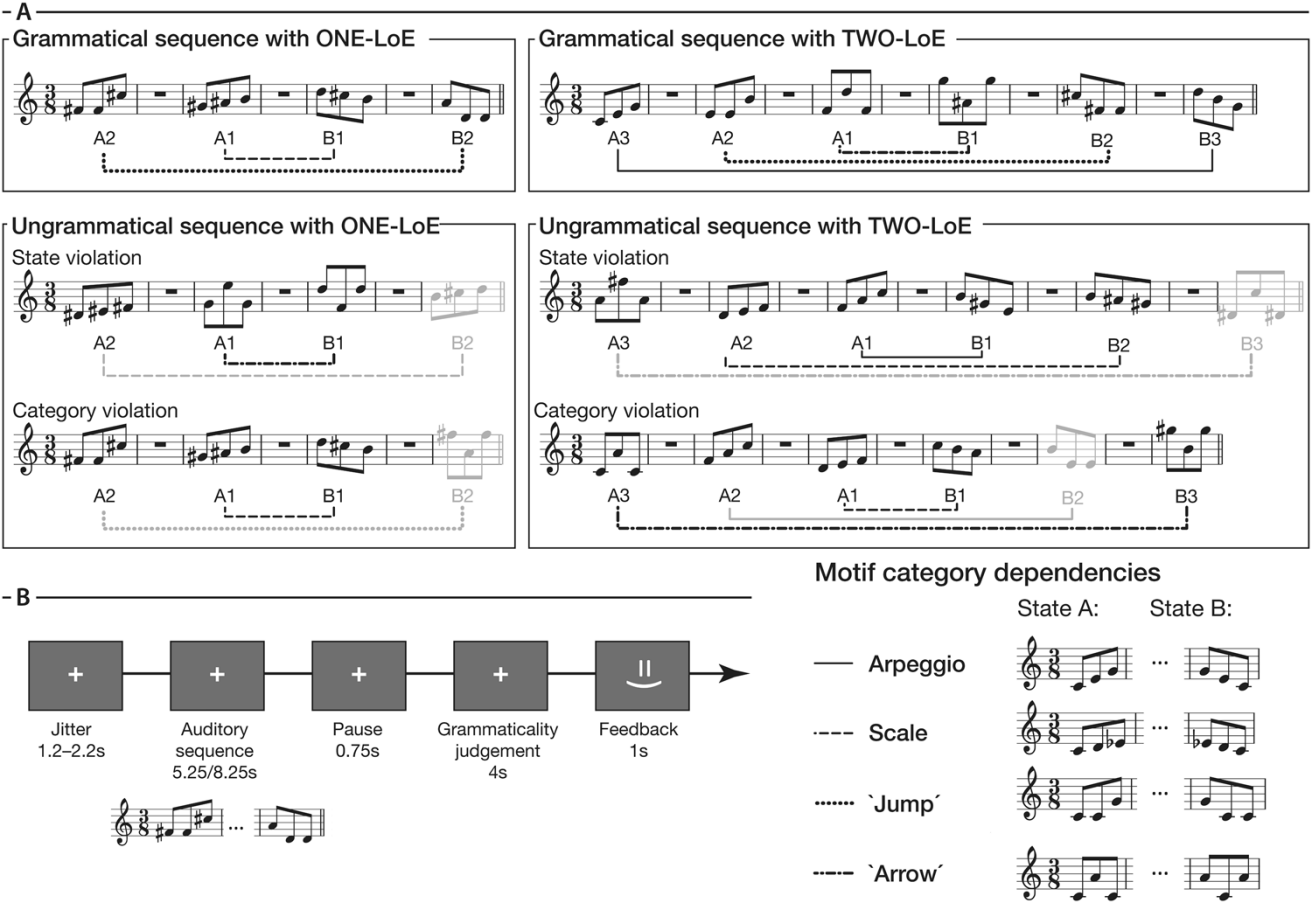
(**A**) Example stimuli. Auditory musical sequences were formed by concatenating three-tone motifs according to the nested atonal grammar A_n_A_n−1_…A_1_B_1_…B_n−1_B_n_. Each motif belonged to one of two states (**A**,**B**), and one of four categories (see lower right, and also Table 1). Categories of A-motifs were randomly concatenated without replacement and were matched by the B-motifs in reverse order. The sequences were manipulated along two factors: GRAMMATICALITY (grammatical vs. ungrammatical) and LEVEL OF EMBEDDING (ONE-LoE (*n* = 2) vs. TWO-LoE (*n* = 3)). Ungrammatical sequences contained exactly one violated B-motif for which its state or category (but not both) was interchanged. Audio versions of the exemplar sequences can be found as Supplementary Audio clips SA1–6. (**B**) Experimental paradigm. Musicians discriminated the grammaticality of 144 novel nested atonal musical sequences, equally divided between combinations of the factors GRAMMATICALITY (grammatical vs. ungrammatical) and LEVEL OF EMBEDDING (ONE-LoE vs. TWO-LoE), during fMRI scanning. Each trial began with a fixation cross at the centre of the screen, and an auditory sequence was presented after a jitter. The symbols ‘Y’ and ‘N’ then respectively appeared to the lower left and right of the fixation cross (pseudo-randomised across trials) and participants were given a 4s-time window to judge the grammatically of the presented sequence. Feedback was then given and a new trial ensued. The nested grammar was previously acquired in a behavioural session around 3.5 weeks before the fMRI experiment.

Auditory sequences of the nested structure A_n_A_n−1_…A_1_B_1_…B_n−1_B_n_ (see Fig. 1A) were generated by concatenating two (ZERO-LoE; structure A_1_B_1_), four (ONE-LoE; structure A_2_A_1_B_1_B_2_), or six musical motifs (TWO-LoE; structure A_3_A_2_A_1_B_1_B_2_B_3_). Each motif consisted of three successive isochronous piano tones (duration = 250 ms per tone) and belonged to one of four categories in one of two states (see Fig. 1A, Table 1 and Supplementary Audio clips SA1–6). The motifs of a sequence were randomly concatenated without replacement so that distinct categories in state A preceded the same categories in state B but in reverse order. This prevented participants from using a counting strategy. Ungrammatical sequences that violated this nested structure were introduced by interchanging either the state (so-called state violations) or category (so-called category violations), but not both, of exactly one B-motif. The replaced motif in a category violation was chosen so that the category was not presented before in the sequence. All sequences contained a pause (duration = 750 ms) between each pair of motifs, and the lowest note of each motif in a sequence was uniformly sampled between all twelve tones in the octave between C4 and B4. This was to avoid participants relying solely on tonal information and accomplishing the task based on matching tones they heard. Sequence durations were thus 2.25 s for ZERO-LoE, 5.25 s for ONE-LoE, and 8.25 s for TWO-LoE.

**Table 1:** Relative pitches of the four motif categories in semitones apart.

Motif category	State	
	A	B
Arpeggio	0-4-7	7-4-0
Scale	0-2-3	3-2-0
Jump	0-0-7	7-0-0
Arrow	0-9-0	9-0-9

Note: The lowest pitch of each motif was between C4 and B4, and was unique for each motif in a nested sequence.

Due to the extensive number of unique sequences (see Supplementary Information), ONE-LoE and TWO-LoE sequences were presented only once throughout the entire experiment. Sequences with ZERO-LoE were used without replacement before being reshuffled back into the pool after each session.

### Procedure

The experiment was divided into a training session and a scanning session, which were around 3.5 weeks apart (mean = 25.76 days, SD = 7.20 days); only participants who acquired the musical grammar in the training session participated in the scanning session (76.67% of participants successfully acquired the grammar). The experiment was programmed on Presentation 18.1 (Neurobehavioral Systems, Inc., Berkeley, CA, USA). Auditory stimuli (mono, 44,100 Hz sampling rate, 16 bits per sample) were delivered at a comfortable volume through circumaural headphones (with foam earplugs inserted inside the MRI scanner). White text was shown against a black background on a computer screen or viewed using a mirror attached to the head-coil from a back-projected image. Foam pads were placed to reduce head movement before fMRI scanning.

In the training session, participants were instructed to learn the grammar of a new language and extract the rule underlying the sequences. The session resembled the learning phase by Bahlmann and colleagues^40^ (see Supplementary Information).

In the scanning session (see Fig. 1B), participants discriminated inside the MRI scanner the grammaticality of 144 novel sequences, equally divided between the four combinations of the two factors: GRAMMATICALITY (GRAMMATICAL versus UNGRAMMATICAL) and LEVEL OF EMBEDDING (ONE-LoE versus TWO-LoE). Violations in ungrammatical sequences were counterbalanced for violation type (i.e. state versus category) and occurrence amongst the B positions. Stimuli were presented across six runs with a break (25 s) between each run. Participants were notified visually 5 s before the end of each break. Stimuli were pseudo-randomised such that at most two consecutive stimuli shared the same LEVEL OF EMBEDDING and GRAMMATICALITY. Each trial began with a randomly-jittered fixation cross (1.2 s–2.2 s) at the centre of the screen, followed by the presentation of an auditory sequence. After a short break (0.75 s), the letters *Y* (grammatical) and *N* (ungrammatical) appeared on the lower left and right sides of the fixation cross, for which participants had a 4 s time-window to decide on the grammaticality of the preceding sequence by pressing either the right index or middle finger on an MR-compatible button box; The letter position was pseudo-randomised. Visual feedback was displayed on the centre of the screen (1 s) to motivate performance (see Data analysis) and the next trial ensued. Additionally, 18 grammatical and 18 ungrammatical 0-LoE sequences were presented as filler sequences and were not analysed.

### Data acquisition

Imaging data were collected on a 3 T Magnetom Skyra scanner (Siemens Healthcare, Erlangen, Germany) with a 20-channel head coil. Slices were acquired axially parallel to the AC-PC line for the whole-brain using a gradient EPI sequence (31 slices per volume, slice thickness = 3 mm, inter-slice gap = 1 mm, acquisition order = odd-interleaved ascending, FoV = 192 mm × 192 mm, acquisition matrix = 64 × 64, TR = 2000 ms, TE = 30 ms, flip angle = 90°, bandwidth = 2004 Hz/Px, echo spacing = 0.56 ms). The functional scan time was 42.3 minutes and 1270 volumes were obtained continuously. T1-weighted structural images (voxel size = 1 mm isotropic) of each participant were used to coregister and normalise the functional images to MNI space.

### Data analysis

Data were analysed using MATLAB R2016a (The MathWorks, Inc., Natick, MA, USA), JASP 0.7.5.6 (JASP Team), and R 3.3.3 (The R Foundation for Statistical Computing, Vienna, Austria). To measure how well participants discriminated between GRAMMATICAL and UNGRAMMATICAL nested musical sequences, we applied signal detection theory^52^ to dissociate their behavioural sensitivity in detecting deviants in grammaticality from their response bias using the non-parametric sensitivity measure *A* and associated log-bias *ln*(*b*)^53^. The sensitivity measure gives an estimate of the mean area under the ROC curve, and the dissociation of sensitivity and bias avoids a misrepresentation of performance due to conflated hit rates. A high sensitivity score thus corresponds to a high hit rate *and* a low false positive rate. As we wanted participants to perform accurately, they were not instructed to respond as quickly as possible and reaction times were not analysed.

After checking for normality using the Shapiro-Wilk test, one-sample t-tests were conducted to compare the overall mean sensitivity against chance-level (0.5), and mean bias against 0, as well as paired t-tests to compare mean sensitivity and bias for the two LEVELS OF EMBEDDING separately. Effect sizes were calculated using Cohen’s *d* for correlated samples^54^.

Imaging data were analysed using SPM 12.6685 (Wellcome Trust Centre for Neuroimaging, London, UK). Volumes were slice-timing corrected, realigned to the first volume using rigid-body transformation and motion-susceptibility correction, coregistered to the individual’s structural image, resampled to a voxel size of 3 mm × 3 mm × 4 mm and normalised to MNI space, and smoothed with a FWHM Gaussian kernel 2.5 times the voxel size for preprocessing.

For statistical analyses at the first-level, a voxelwise GLM was estimated for each participant. Each sequence was modelled as a boxcar function of the same stimulus duration and convolved with the canonical HRF. One regressor was used to model correctly-responded sequences for each combination of the two factors: GRAMMATICALITY (GRAMMATICAL versus UNGRAMMATICAL) and LEVEL OF EMBEDDING (ONE-LoE versus TWO-LoE). A regressor modelling all remaining sequences, a regressor indicating volumes within breaks, a regressor to remove finger-press artifacts, and six motion regressors were added as regressors of no interest. Finger presses were modelled using a boxcar function with the response-prompt as onset and reaction time as duration and were convolved with the canonical HRF. A high-pass filter (128 s cut-off) and an autoregressive AR(1) model were applied.

To guarantee that the observed effects on grammaticality were not confounded by serial processing, we additionally estimated a refined model at the subject-level where ungrammatical sequences only consisted of correctly-responded category violations at positions B_2_ and B_3_ *post hoc*. Sequences with violations in position B_1_ or state violations could have been rejected by a strategy which does not require resolving non-adjacent dependencies, as it might have been possible for participants to have detected these violations by only comparing surface features of the violated motif and the preceding motif^55,56^. However, it is unlikely that participants relied solely on serial or local processing to reject these sequences, because (1) they could not have known beforehand the grammaticality of a sequence and position of the violation, and so would have had built a nested representation of the motifs to complete the task in case the sequence was grammatical, and (2) only participants who were proficient in detecting category violations were included in the analysis.

For statistical analyses at the group-level, data were modelled by a 2 × 2 flexible factorial model. We assumed independence at the subject and condition levels, equal variance in the former and unequal in the latter. Significant clusters were identified using an *a priori*-defined voxelwise FWE-corrected threshold of *p* < 0.05 and an extent of four voxels, determined by rounding above the expected cluster size estimated from the smoothness of the SPM based on Gaussian random-field theory^57^. Anatomical locations were identified using the MNI2TAL tool^58^ and SPM Anatomy Toolbox 2.2c^59^.

We turned to a psychophysiological interaction (PPI) analysis to explore the extent to which brain regions implicated in the current study are functionally related. In brief, PPI measures the change in functional connectivity between two regions under different experimental contexts^60^. This allows us to infer any (undirected) flow of task-relevant information between two brain regions. Given our factorial design, we carried out a generalised PPI analysis^61^ as opposed to a traditional PPI analysis^62^. Generalised PPI is more suited to our experiment as the model includes all psychological factors and hence spans the entire experimental space.

Seed regions were defined by drawing spheres (radii = 4.5 mm, chosen to avoid overlap between the regions of interest) around the maxima of each significant cluster of the refined model. They were then multiplied with the group mask to ensure that each seed region only included brain voxels present in all participants. We examined the task-modulated functional connectivity between each seed region and the remaining clusters across the four conditions by comparing the gPPI regressors of each subject in a 2 × 2 flexible factorial model at the group-level. An explicit mask of the seed regions was applied during the estimation of the second-level model, and we adopted the same statistical threshold as before. The analysis was performed using the gPPI toolbox 13.1^61^.

To further examine whether psychophysiological interactions predicted behavioural performance, we additionally correlated the mean difference in beta estimates of each significant PPI cluster with participants’ overall sensitivity in detecting grammatical violations. The beta estimates of each cluster were extracted using MarsBaR 0.44^63^.

Data analysed during this study are available from the corresponding author upon reasonable request.

## Results

### Behavioural results

**Figure 2:**
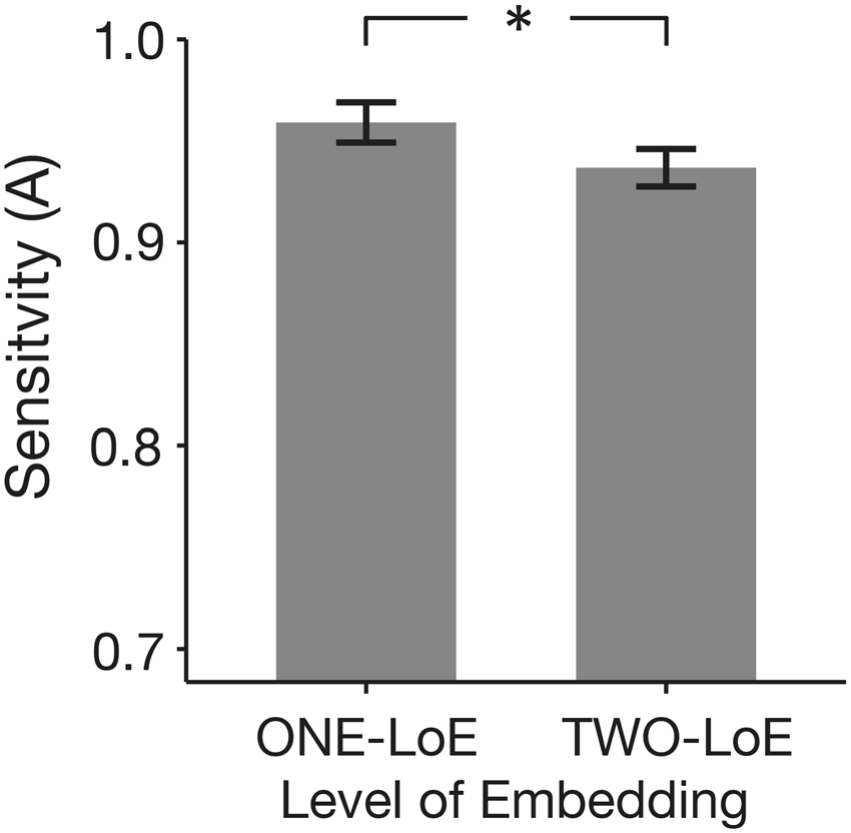
Behavioural results. Sensitivity in discriminating the grammaticality of nested musical sequences was significantly higher for ONE compared to TWO levels of embedding. Error-bars indicate standard error. *Indicates *p* < 0.05.

Participants’ overall sensitivity (M = 0.95 (out of a maximum score of 1), SD = 0.04, range = 0.88–1.00) in discriminating between grammatical and ungrammatical nested musical sequences was significantly above chance (*t*(16) = 51.92, *p* = 1.44 × 10^−19^, Cohen’s *d* = 12.59) and correlated with the number of years of training in their most experienced instrument (*r* = 0.63, *p*(*corrected*) = 0.03, see Figure S1 and Supplementary Information), although the response bias (M = −0.19, SD = 0.17) indicated a significant bias towards judging a sequence as grammatical (*t*(16) = −4.52, *p* = 3.47 × 10^−4^, Cohen’s *d* = −1.10). The mean sensitivity for ONE-level of embedding (LoE) sequences was also significantly higher than TWO-LoE sequences, but the difference in bias between the two levels of embedding was not significant (see Fig. 2 and Table 2).

**Table 2:** Mean sensitivity and bias in detecting violations in nested musical sequences.

	LEVEL OF EMBEDDING		*t*(16)	*p*-value	Cohen’s *d*
	ONE	TWO			
Sensitivity	0.96 (0.04)	0.94 (0.04)	2.73	0.02*	0.66
Bias	−0.16 (0.23)	−0.22 (0.17)	1.22	0.24	0.30

Notes: Values inside brackets indicate standard deviation. Maximum possible sensitivity score is 1; A negative bias indicates a tendency to judge a sequence as grammatical; *p < 0.05.

### Imaging results

**Figure 3:**
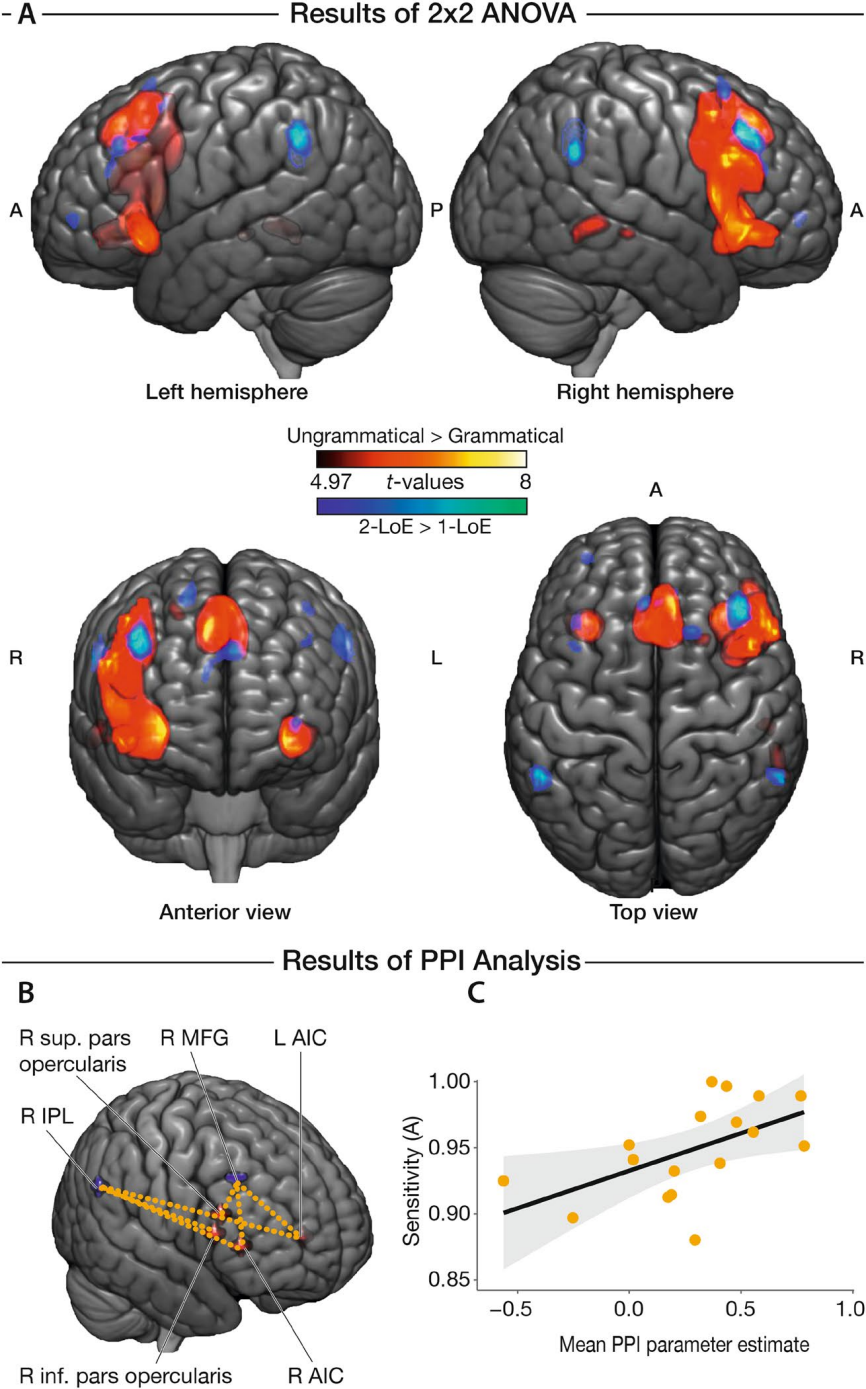
(**A**) Whole-brain activations for main effects of grammaticality and level of embedding (LoE) on discriminating the grammaticality of nested musical sequences. The contrast UNGRAMMATICAL > GRAMAMTICAL yielded significant clusters (red) in the right inferior frontal gyrus, right middle frontal gyrus, bilateral anterior insular cortices, the pre-supplementary motor area, and the right posterior middle temporal gyrus. Contrasting sequences with TWO-LoE > ONE-LoE yielded significant clusters (blue) bilaterally in the middle frontal gyrus and inferior parietal lobule. Reported clusters were corrected for multiple comparisons voxel-wise at a threshold of *p* < 0.05 and an extent of 4 voxels. (**B**) PPI analysis on significant clusters of the refined model where ungrammatical sequences only included non-local category violations. Using the refined model, activity was observed in the right pars opercularis, right pars triangularis, and bilateral anterior insular cortices for the contrast UNGRAMMATICAL > GRAMAMTICAL (seed regions in red), and the right middle frontal gyrus and right inferior parietal lobule for the contrast TWO-LoE > ONE-LoE (seed regions in blue). Dotted lines indicate significantly-increased functional connectivity between seed regions of significant clusters in the experimental context of UNGRAMMATICAL sequences compared to GRAMMATICAL. Results were corrected for multiple comparisons voxel-wise at a threshold of *p* < 0.05 and an extent of 4 voxels. (**C**) Positive correlation between sensitivity in discriminating the grammaticality of nested musical sequences and increase in task modulated functional connectivity (*r* = 0.55, *p* = 0.03, 1-tailed test; corrected). Shaded region is the 95% confidence band of the linear regression line.

Distinct clusters of significant BOLD-response differences (see Fig. 3A and Table 3) were evaluated using SPM *t*-contrasts at the whole-brain level for both main effects of GRAMMATICALITY and LEVEL OF EMBEDDING. For the main effect of GRAMMATICALITY (UNGRAMMATICAL > GRAMMATICAL), we found a cluster of increased BOLD response with maxima in the right inferior frontal gyrus (IFG; pars opercularis, triangularis, and orbitalis), right middle frontal gyrus (in the dorsolateral prefrontal cortex), and right anterior insular cortex (AIC). We additionally identified clusters in the pre-supplementary motor area (pre-SMA), right dorsal premotor cortex, and left anterior insular cortex (AIC). The reverse contrast (GRAMMATICAL > UNGRAMMATICAL) yielded increased responses in the bilateral ventromedial prefrontal cortex (vmPFC).

**Table 3:** Significant clusters showing differential BOLD responses with respect to the GRAMMATICALITY and LEVEL OF EMBEDDING (LoE) of nested musical sequences.

Anatomical region	BA	Cluster size (voxels)	MNI-coordinates			*t*-value
			X	Y	Z	
**UNGRAMMATICAL** > **GRAMMATICAL**						
R anterior insular cortex (AIC)		663	33	23	−2	8.42
R pars opercularis	44		51	17	10	8.05
R pars triangularis	45		48	23	26	7.12
R pars orbitalis	47		45	32	−6	7.46
R middle frontal gyrus	8		45	14	50	7.01
L/R Pre-supplementary motor area (pre-SMA)	8	234	6	32	46	8.78
			−3	23	54	7.18
L anterior insular cortex (AIC)		72	−30	20	−6	7.89
R middle temporal gyrus	21	24	57	−43	−2	5.62
	21/22	7	54	−25	−6	5.41
R dorsal premotor cortex	6	9	21	14	50	5.40
**GRAMMATICAL > UNGRAMMATICAL**						
L/R ventromedial prefrontal cortex (vmPFC)	11	80	−12	41	−10	7.22
			6	41	−14	6.27
**TWO-LoE > ONE-LoE**						
R middle frontal gyrus	9	42	42	29	42	6.79
L inferior parietal lobule (IPL)	39/40	31	−54	−49	42	6.31
R inferior parietal lobule (IPL)	39	23	60	−49	30	6.26
Pre-supplementary motor area (pre-SMA)	8	21	−6	32	34	5.85
R dorsal premotor cortex	6	10	18	20	58	5.73
L middle frontal gyrus	9	8	−36	23	38	5.23
	6/8	6	−39	14	54	5.33
L frontal pole	10	5	−33	53	−2	5.32
**ONE-LoE > TWO-LoE**						
L supplementary motor area (SMA)	6/4	151	−36	−19	58	6.90
L premotor cortex	6	27	−6	2	54	6.03
L superior temporal gyrus	41	15	−54	−19	6	5.59
R superior temporal gyrus	22	14	66	−22	2	6.45

Notes: Reported clusters have a minimum cluster size of 4 voxels and were corrected for multiple comparisons voxel-wise using a threshold of *p* < 0.05. L – left hemisphere; R – right hemisphere; *t*-contrasts compared are shown in bold; Anatomical regions within each cluster are at least 4 mm apart and are ranked according to decreasing *t*-values.

For the main effect of LEVEL OF EMBEDDING (TWO-LoE > ONE-LoE), we observed bilaterally clusters in the inferior parietal lobule (IPL), middle frontal gyrus, dorsal premotor cortex, pre-supplementary motor area, and left frontal pole. For the reverse contrast (ONE-LoE > TWO-LoE), we identified clusters in the bilateral middle superior temporal gyri, and left premotor cortex.

No supra-threshold clusters were yielded for the interaction contrast (even at a more lenient cluster-wise FWE-corrected threshold of *p* < 0.05).

Results of the refined model which only contained category violations in ungrammatical sequences (see Materials and Methods) were furthermore analogous to the original model. The GRAMMATICALITY contrast (UNGRAMMATICAL > GRAMMATICAL) yielded four significant clusters with maxima in the right anterior insular cortex (coordinates in MNI space: [33, 26, 2]), right pars opercularis ([45, 17, 10]), right pars triangularis ([48, 23, 22]), and left anterior insular cortex (AIC; [−33, 20, −6]), whilst the LEVEL OF EMBEDDING contrast (TWO-LoE > ONE-LoE) yielded two significant clusters with maxima in the right inferior parietal lobule (IPL; [60, −49, 30]), and the right middle frontal gyrus ([42, 29, 42]). No significant clusters in the reverse and interaction contrasts were observed at the voxel-corrected statistical threshold (although a more-lenient cluster-corrected threshold nonetheless yielded significant clusters in the same regions as in the original model).

### Psychophysiological Interaction (PPI) analysis

For the main effect of GRAMMATICALITY (UNGRAMMATICAL > GRAMMATICAL), we found significant psychophysiological interactions (see Fig. 3B and Table 4) between the right IPL (as seed) and the right pars opercularis and bilateral AIC. Only the psychophysiological interaction of the right IPL on the right AIC significantly correlated with participants’ overall sensitivity in discriminating between grammatical and ungrammatical sequences (see Fig. 3C, *r* = 0.55, *p* = 0.03, one-tailed test; corrected using Holm’s method across all significant clusters with the same seed region).

**Table 4:** Maxima of clusters showing increased psychophysiological interactions during the experimental context of UNGRAMMATICAL versus GRAMMATICAL nested sequences.

Anatomical region	BA	Cluster size (voxels)	MNI-coordinates			*t*-value
			X	Y	Z	
**Seed: R inferior parietal lobule** (**IPL**)						
R anterior insular cortex (AIC)		9	30	29	2	4.34
L anterior insular cortex (AIC)		7	−30	23	−6	4.11
R pars opercularis*	44	9	51	20	22	3.87
**Seed: R middle frontal gyrus**						
R anterior insular cortex (AIC)		9	30	26	2	4.63
L anterior insular cortex (AIC)		6	−30	20	−6	4.55
R pars triangularis/ inferior frontal sulcus	45/9	7	48	20	22	3.91

Notes: Reported clusters were corrected for multiple comparisons voxel-wise using a statistical threshold of *p* < 0.05 and an extent of 4 voxels. *This cluster had another local maxima 8.49 mm apart. L – left hemisphere; R – right hemisphere.

We also found significant interactions between the right middle frontal gyrus (as seed) and the right pars triangularis/inferior frontal sulcus and bilateral AIC with respect to GRAMMATICALITY Other combinations of contrasts and seed regions did not yield significant results.

## Discussion

The present experiment aimed to uncover the functional neural basis underlying the human ability to process non-local dependencies – a key feature of hierarchical structures – in music. By independently manipulating the grammaticality and auditory tonal working memory demands of nested atonal musical sequences, we found that grammatical violations of nested musical dependencies led to increased BOLD responses in the right inferior frontal gyrus (IFG) and bilateral anterior insular cortices (AIC), whilst increased auditory working memory demands led to enhanced responses in the bilateral middle frontal gyri (MFG) and inferior parietal lobules (IPL). This result confirms our hypothesis that the inferior frontal gyrus – especially the right homologue of Broca’s area – is involved in processing non-local dependencies in music. Modulations in functional connectivity between these two distinct functional networks were also associated with discriminating between grammatical and ungrammatical nested sequences. In particular, the task-modulated connectivity between the right AIC and right IPL predicted behavioural performance. These suggest that resolving non-local dependencies in music requires the interplay between brain regions involved in processing hierarchical structures in music and brain regions involved in tonal working memory.

Our findings provide the first evidence that engagement of the right IFG in the neurocognition of music reflects processing of nested non-local dependencies based on internal knowledge of the grammatical rules of musical syntax. Importantly, our refined analysis suggests that the posterior right IFG is sensitive to violations of exclusively non-local dependencies in the absence of any local violation between immediate-adjacent elements. This clarifies prior work that had observed right-lateralised IFG activity (e.g. Koelsch *et al*., 2005, 2002) but employed paradigms that confounded hierarchical and local irregularity (e.g. a chord other than the tonic following a dominant seventh chord at the end of a chord sequence), such that observed IFG activity could have resulted from to local (serial) processing alone.

Participants’ post-experiment reports moreover suggest that the nested non-local dependencies were processed hierarchically. When prompted to explain what the underlying rule for the sequences were in an open-ended manner, all participants used terms such as ‘mirror’ (seven participants), ‘symmetry’ (four participants), ‘tree’ (one participant), or that the second half is the same as the first but in reversed/inverted order (five participants), to explain how the sequences were arranged. Given that each test sequence was unique and participants were never told what the generative rule was (see Materials and Methods), this demonstrates that participants were able to abstract surface features of the stimuli into a syntactic rule that describes their structure. This representation is moreover hierarchical because the A-motifs (e.g. A_3_A_2_A_1_) are grouped into a superordinate set that is mirror-transformed into another superordinate set containing the B-motifs (e.g. B_1_B_2_B_3_) in the second half. Importantly, participants exploited their understanding of the mirror-symmetric rule to accomplish the task. Participants actively predicted what the next motif could be in each sequence and compared their predictions with the incoming motif. A sequence was deemed grammatical if all predictions were met, and ungrammatical otherwise. This required participants to maintain multiple nested dependencies in parallel and in the correct order, as well as to monitor which superordinate class a motif belonged to (i.e. whether the upcoming motif would belong to the first or second half of a sequence).

Our finding of the right IFG in processing nested non-local dependencies in music thus supports the view that Broca’s area and its right homologue are involved in processing hierarchical structure, although with different hemispheric-weightings across various cognitive domains. While processing linguistic syntax is weighted towards the left hemisphere, we suggest that processing hierarchical structure in music is weighted towards the right: Previous studies have shown that the left IFG is sensitive to violations in nested artificial phoneme sequences^64^, and elicited increased activity for nested hierarchical sequences compared to serial non-hierarchical verbal sequences^30,40,64,65^. The pars opercularis of the left IFG in particular showed functional specificity to syntactic information in language^29^. Therefore, the observed activity in the right IFG – particularly the pars opercularis as highlighted in our refined analysis – likely reflects mechanisms which involve constructing hierarchical representations of the incoming acoustic information^66^.

The present data therefore raise the possibility that at least some aspects of hierarchical processing may rely on lateralised, domain-selective neuronal populations. Despite proposals for an interaction between syntactic processing in music and language^32,34,67,68^, our findings are more in line with the proposal that representing hierarchical dependencies could differ in music and language^69^, and thus calls for the existence of domain-specific resources in parsing the hierarchical dependencies. Musso and colleagues^70^, for example suggest that processing syntax in music and language is highly differentiated within the left IFG, but more generally engages in a dual-stream system that connects left frontal, parietal, and temporal regions. Our findings therefore motivate further studies on clarifying the extent to which music and language share common neural resources, and to what extent the observed effects in the right IFG pertain to syntactic processing specifically, or to general mechanisms such as attention^71^ or cognitive control^72,73^.

Nevertheless, our proposed clear-cut separation between the left IFG processing linguistic syntax and the right IFG processing musical syntax is only tentative: In light of the recent controversy on inflated false-positive rates in fMRI studies^74^, we adopted a conservative correction for multiple comparisons at the expense of statistical sensitivity – significantly increased BOLD responses to violations in musical syntax were indeed also observed in the left IFG at a more lenient cluster-wise corrected statistical threshold. Second, the participants of our study were trained musicians – a group shown to exhibit increased grey-matter volume in the right IFG^75^, higher fibre-tract volumes between the right IFG and the temporal lobe^76^, and differences in BOLD-response patterns^77–79^, compared to non-musicians. Alternatively, effects of lateralisation could have been driven by the idiosyncratic differences in experimental stimuli. For example, phonemes in speech are typically much shorter in duration than musical notes. It has been argued that auditory information is extracted at different timescales between the two hemispheres^80^, and that the left hemisphere is more specialised towards processing temporal features whilst the right towards spectral features of the auditory stream^46,81^. This mechanism could also explain how musical stimuli does not consistently engage the right or left IFG.

Furthermore, the role of hierarchy in resolving nested dependencies in artificial grammar studies has also been challenged. It has been argued that sequential processing is more parsimonious^82^, and that there is no intrinsic super-/subordinate relation across elements in a sequence^83^. In light of this, we are cautious in interpreting how processing nested non-local dependencies in our sequences extended towards hierarchical processing of music, and only suggest the role of the right IFG in the hierarchical processing of music based on participants’ self reports.

In addition to our experimental manipulation of grammaticality in a nested musical syntax, we also manipulated the demands of auditory tonal working memory by varying the number of embedding levels. When contrasting sequences with two levels of embedding against one, we observed decreased behavioural performance and increased BOLD responses in the inferior parietal lobule (IPL) and the middle frontal gyrus (MFG) of the dorsolateral prefrontal cortex (dlPFC). These effects likely reflect the established role of the IPL and dlPFC in tonal working memory, given the additional dependency induced by an additional pair of motifs to be held in memory. In accordance with this interpretation, activity in the MFG and IPL were shown to be involved in same/difference tasks in musical melodies^46,84^, and n-back tasks using chord sequences^85^. Similar responses have also been observed during the maintenance of pitch information^43,45,46,86^.

However, because motifs were matched with their transposed conjugates, participants had to maintain interval relations between the motifs and not their absolute pitch classes. Consequently, the contrast between sequences with two levels of embedding against one also reflects manipulation – on top of encoding and maintenance – of the incoming acoustic signals in tonal working memory. Previous studies have suggested that dlPFC activity may reflect modulatory top-down control signals to information represented elsewhere in the neocortex^87–90^, whilst the IPL was shown to be involved in integrating acoustic melodic information within a tonal context^91^, as well as comparing original and transposed melodies^84,92^. According to state-based models of working memory^87,93,94^, information held in working memory are distributed across the cortex and represented in increasing levels of abstractness: from relatively raw and unprocessed in the sensory cortices to highly abstract in the frontal cortex^93^. The observed increased BOLD response in the dlPFC may therefore reflect increased attention in maintaining the additional pair of motifs that is represented in a transposed and processed format in the IPL (please also see the Supplementary Information for the discussion on some brain areas for which we did not have clear *a priori* hypotheses).

Extending the functional main effects that we have just discussed, our psychophysiological interaction (PPI) analysis suggests that the behavioural sensitivity to violations of musical syntax requires the interaction of a system that processes nested hierarchical information (i.e. IFG and AIC), and an auditory working memory system (i.e. MFG and IPL). We thus propose that the ability to resolve nested hierarchies in music depends on how salient the constituent musical elements are represented in working memory. With this interpretation, BOLD response modulation in the AIC reflects participants’ awareness to violations that motivates the appropriate motor preparation and response. In addition to the involvement of the AIC in music cognition^22,25,27,43,45,46,95,96^, the right AIC is a key node of the salience network that detects behaviourally relevant stimuli^97^, supports the translation of affective signals into specific actions^98,99^ and is associated with perceptual decision-making, interoception, and emotional awareness^98,100–103^. A second mathematically equivalent interpretation of our PPI result is that processing violations in musical syntax instead drives functional connectivity between the IFG and AIC. We suggest that this is less plausible, because discriminating between grammatical and ungrammatical sequences required similar demands in maintaining the musical motifs in tonal working memory.

A limitation in comparing sequences with two levels of embedding against one is the inclusion of an extra pair of motifs that necessitates a longer sequence duration, which confounds distance and item effects. Although this limitation is present in other studies using similar artificial grammar-like nested sequences^64,104^, the effects of length were typically not observed, possibly because the differences in duration in those studies were around 1 s compared to 3 s in the current experiment. This difference could explain the supposed activity of the supplementary motor area when comparing sequences with one level of embedding against two – which most likely reflects motor preparation for the ensuing button response after the stimuli. Moreover, although we were able to infer from participants’ reports that they were sensitive to the order and pairing between A and B motifs, further studies could for example include a reordering violation (e.g. A_3_A_2_A_1_B_3_B_1_B_2_) to verify that participants were indeed sensitive to the order of motifs presented.

In conclusion, processing hierarchical structure in music may involve two functionally segregated but nonetheless interacting systems in the right hemisphere: the IFG and AIC in resolving nested non-local dependencies between musical elements, and the MFG and IPL in auditory tonal working memory. The resemblance of the right-lateralised activation profile in processing non-local dependencies in music with the left-hemispheric system in language moreover suggests that processing hierarchical structures in music and language involves qualitatively similar mechanisms subserved by domain-specific neural subpopulations.

## Electronic supplementary material

Supplemental Information

Supplementary Audio SA1

Supplementary Audio SA2

Supplementary Audio SA3

Supplementary Audio SA4

Supplementary Audio SA5

Supplementary Audio SA6
