# Supplementary material for: The right inferior frontal gyrus processes nested non-local dependencies in music
Source: Sci Rep. 2018 Feb 28;8:3822. doi: 10.1038/s41598-018-22144-9 (PMC5830458; doi:10.1038/s41598-018-22144-9)
Supplement: Supplementary file 1 — Supplemental Information [file 41598_2018_22144_MOESM1_ESM.pdf]

Supplementary Information to

The right inferior frontal gyrus processes nested non-local dependencies in music

Vincent K.M. Cheung (cheung@cbs.mpg.de) [*, a]

Lars Meyer (lmeyer@cbs.mpg.de) [a]

Angela D. Friederici (friederici@cbs.mpg.de) [a]

Stefan Koelsch (koelsch@cbs.mpg.de) [b, a]

[a] Department of Neuropsychology, Max Planck Institute for Human Cognitive and Brain Sciences, Leipzig, Germany

[b] Department of Biological and Medical Psychology, University of Bergen, Bergen, Norway

[*]Corresponding author

Supplementary Materials and Methods

Stimuli generation

Auditory versions of each motif were generated using MIDI file tools for MATLAB (Ken Schutte, https://github.com/kts/matlab-midi) in MATLAB R2015b (The MathWorks, Inc., Natick, MA, USA) and rendered into wave files using a Yamaha Disklavier Pro SoundFont (Roberto Gordo Saez, Zenph Studios) in FluidSynth 1.1.5 (FluidSynth team, http://www.fluidsynth.org/), then converted from stereo to mono, and normalised using Audacity 2.0.6 (Audacity Team, https://sourceforge.net/projects/audacity/).

Total number of possible sequences

Given a total of $12!/(12 – 2n)!$ pitch arrangements and $4!/(4 – n)!$ motif category choices, where n denotes the number of A-motifs, there were 528 unique grammatical sequences for ZERO-Levels of Embedding (LoE), 142,560 for ONE-LoE, and 15,966,720 for TWO-LoE. Moreover, as violations could occur at any B-motif, the total number of state-violated sequences was n times the number of grammatical sequences, whilst the total number of category-violated sequences was $n(4 – n)$ times the number of grammatical sequences because of the additional $(4 – n)$ motif category substitutes.

Training procedure

Sequences with ZERO-LoE were trained in level one, ONE-LoE in level two, and TWO-LoE in level three. Once participants advanced beyond level three, level four was presented where grammatical and ungrammatical sequences with ONE-LoE or TWO-LoE were presented in a counterbalanced fashion. Levels were subdivided into blocks of 10 training- and 10 test-sequences, and participants progressed to the next level once they correctly discriminated the

grammaticality of at least 9 out of 10 test sequences in two consecutive blocks. Participants who reached level four were invited to the scanning session. The experiment was over if the participant did not progress after 20 blocks. Each block started with a visual instruction (3 s) to attend to the training sequences. A trial began by displaying the sequence number (1.2 s), followed by the playback of a grammatical sound sequence and the replacement of the text by a fixation cross at the centre of the screen. Afterwards, another visual instruction was given (3 s) to judge the grammaticality of 10 subsequent test sequences, of which 5 were ungrammatical. Grammaticality of the test sequences was pseudo-randomised, such that there could only be at most two successive presentations of sequences with identical grammaticality, and the two types of violations for ungrammatical sequences were roughly balanced. Following a fixation cross (1.2 s) and the ensuing stimulus, participants were given a time window (3 s) to respond to a question (*Did the sequence correspond to the rule?*) by selecting either *Yes* (left arrow key) or *No* (right arrow key) on a computer keyboard. Key assignment was counterbalanced between participants. Visual feedback in the form of a smiley or sad emoticon was given (1.2 s), followed by a pause (0.75 s) and the next trial ensued.

To acquaint participants with the new response format (where the position of the grammatical response was now variable), participants heard 10 grammatical sequences with ZERO-, ONE-, and TWO-LoE and practiced the task on 30 new sequences prior to fMRI scanning.

Supplementary Results

Correlation of behavioural data

We also correlated our behavioural results with the number of days apart between sessions, reverse digit span (using PEBL 0.14 [1]), general musical sophistication, and the number of years of training in participants' most experienced instrument using Pearson's correlation. These participant-characteristics constituted the family for which we applied Holm's method [2] to correct for multiple comparisons.

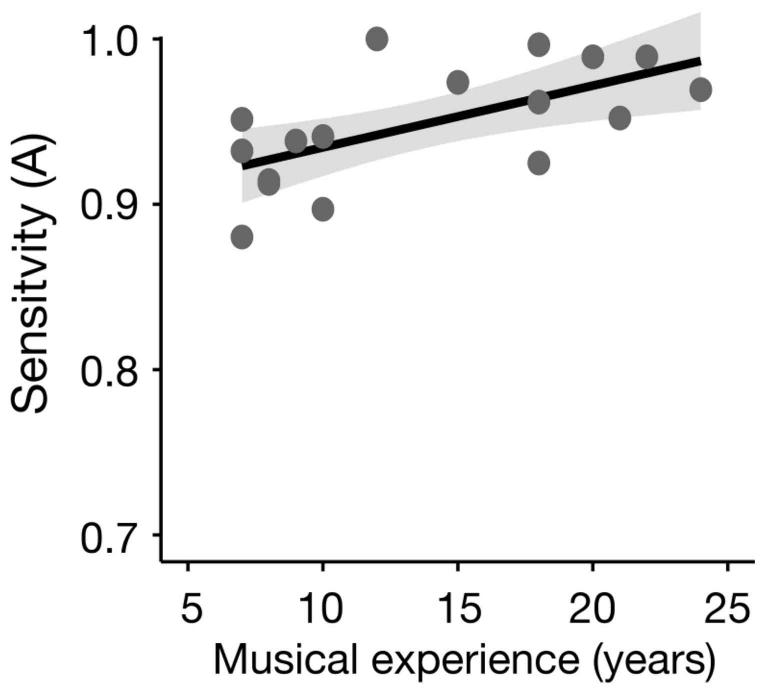

Figure S1: Positive correlation between overall sensitivity and number of years of training in participants' most-experienced instrument (r = 0.63, p = 0.03 (corrected)). Shaded region is the 95% confidence interval band of the linear regression line.

Supplementary Discussion

Grammaticality processing: dorsolateral PFC and pre-SMA

As opposed to the functional activity of the right IFG (see main discussion), the current literature suggests that the involvement of the dorsolateral prefrontal cortex (dlPFC), pre-supplementary motor area (pre-SMA), and the anterior insular cortex (AIC) in grammaticality may rather be due to their complementary roles in cognitive control (cf. Cole and Schneider, 2007). Cognitive control processes dynamically assign mental resources to modify and control behaviour according to one's goals and intentions [4,5]. In the context of the present experiment, the dlPFC and pre-SMA may respectively supplement violation detection by enabling participants to (1) actively maintain task-relevant information, and (2) prepare and initiate the appropriate motor response. As in the main discussion, cognitive control signals generated by the dlPFC are said to activate representations of the incoming motifs, which can then be compared to determine whether the nested dependencies have been correctly resolved. Second, pre-SMA activity has been implicated in the integration of sequential information in speech and music [6,7], but also in other domains [8], into higher-order schemas. Alternatively the pre-SMA could reflect the different brain states pertaining to the two grammaticality conditions in preparation for the subsequent behavioural response, as shown through its implication in the timing of self-initiated motor responses [9] and the maintenance of abstract contingencies in preparation for the selection of concrete motor responses [10–12]. Importantly, pre-SMA activity cannot reflect the actual motor response, as the position of the correct finger response was pseudo-randomly assigned across trials.

Grammaticality processing: middle temporal gyrus

The increased activity in the right posterior middle temporal gyrus (MTG) for ungrammatical against grammatical nested sequences is possibly due to the processing of meaning information. Although the observed co-activation of the MTG and the IFG echoes previous studies on violations to tonal harmony [13] and musical expectancy [14,15], the MTG has typically been associated with the processing and storage of semantic information in music [16–18] and language [19–22]. On one hand, participants may have attributed semantic information to the nested sequences by assigning a unique internal representation for each motif to reliably discriminate between grammatical and ungrammatical sequences. On the other hand, music theorists (e.g. Meyer [23]) have traditionally argued that musical meaning arises from the interplay of tension and relaxation across multiple levels of musical structure. Grammatical and ungrammatical nested sequences in the current experiment may have thus conveyed different musical meaning information through their respective fulfilment and deviation of predictions, as suggested from their behavioural data. The likelihood that the processing of nested hierarchical dependencies in music involves the dynamic integration of syntactic information in the IFG with semantic information in the MFG is increased by evidence on the regions' strong structural and functional tractography [24].

Grammaticality processing: ventromedial prefrontal cortex

The ventromedial prefrontal cortex (vmPFC) was the only region that showed increased BOLD response to grammatical nested musical sequences compared to ungrammatical. While this finding was unexpected, the vmPFC has been proposed to be involved in decision-making by encoding and updating the value of alternative outcomes [25–27]. We thus speculate that increased vmPFC activity for grammatical sequences reflects the confirmation reward received when the incoming motifs match participants' predictions.

Supplementary References

1. Mueller, S. T. & Piper, B. J. The Psychology Experiment Building Language (PEBL) and PEBL Test Battery. *J. Neurosci. Methods* **222,** 250–259 (2014).
2. Holm, S. A Simple Sequentially Rejective Multiple Test Procedure. *Scand. J. Stat.* **6,** 65–70 (1979).
3. Cole, M. W. & Schneider, W. The cognitive control network: Integrated cortical regions with dissociable functions. *Neuroimage* **37,** 343–360 (2007).
4. Fan, J. An information theory account of cognitive control. *Front. Hum. Neurosci.* **8,** 1–16 (2014).
5. Badre, D. Cognitive control, hierarchy, and the rostro-caudal organization of the frontal lobes. *Trends Cogn. Sci.* **12,** 193–200 (2008).
6. Lima, C. F., Krishnan, S. & Scott, S. K. Roles of Supplementary Motor Areas in Auditory Processing and Auditory Imagery. *Trends Neurosci.* **39,** 527–542 (2016).
7. Hertrich, I., Dietrich, S. & Ackermann, H. The role of the supplementary motor area for speech and language processing. *Neurosci. Biobehav. Rev.* **68,** 602–610 (2016).
8. Cona, G. & Semenza, C. Supplementary motor area as key structure for domain-general sequence processing: A unified account. *Neurosci. Biobehav. Rev.* **72,** 28–42 (2017).
9. Soon, C. S., He, A. H., Bode, S. & Haynes, J.-D. Predicting free choices for abstract intentions. *Proc. Natl. Acad. Sci. U. S. A.* **110,** 6217–22 (2013).
10. Petit, L., Courtney, S. M., Ungerleider, L. G. & Haxby, J. V. Sustained Activity in the Medial Wall during Working Demory delays. *J. Neurosci.* **18,** 9429–9437 (1998).
11. Bunge, S. a, Kahn, I., Wallis, J. D., Miller, E. K. & Wagner, A. D. Neural circuits subserving the retrieval and maintenance of abstract rules. *J. Neurophysiol.* **90,** 3419–3428 (2003).

12. Cunnington, R., Windischberger, C., Robinson, S. & Moser, E. The selection of intended actions and the observation of others' actions: A time-resolved fMRI study. *Neuroimage* **29,** 1294–1302 (2006).

13. Koelsch, S., Fritz, T., Schulze, K., Alsop, D. & Schlaug, G. Adults and children processing music: An fMRI study. *Neuroimage* **25,** 1068–1076 (2005).

14. Tillmann, B. *et al.* Cognitive priming in sung and instrumental music: Activation of inferior frontal cortex. *Neuroimage* **31,** 1771–1782 (2006).

15. Tillmann, B., Janata, P. & Bharucha, J. J. Activation of the Inferior Frontal Cortex in Musical Priming. *Ann. N. Y. Acad. Sci.* **999,** 209–211 (2003).

16. Groussard, M. *et al.* The neural substrates of musical memory revealed by fMRI and two semantic tasks. *Neuroimage* **53,** 1301–1309 (2010).

17. Koelsch, S. *et al.* Music, language and meaning: brain signatures of semantic processing. *Nat. Neurosci.* **7,** 302–307 (2004).

18. Platel, H., Baron, J. C., Desgranges, B., Bernard, F. & Eustache, F. Semantic and episodic memory of music are subserved by distinct neural networks. *Neuroimage* **20,** 244–256 (2003).

19. Ni, W. *et al.* An event-related neuroimaging study distinguishing form and content in sentence processing. *J. Cogn. Neurosci.* **12,** 120–133 (2000).

20. Binder, J. R., Desai, R. H., Graves, W. W. & Conant, L. L. Where is the semantic system? A critical review and meta-analysis of 120 functional neuroimaging studies. *Cereb. Cortex* **19,** 2767–2796 (2009).

21. Lau, E. F., Phillips, C. & Poeppel, D. A cortical network for semantics: (de)constructing the N400. *Nat. Rev. Neurosci.* **9,** 920–933 (2008).

22. Friederici, A. D. The Brain Basis of Language Processing: From Structure to Function. *Physiol. Rev.* **91,** 1357–1392 (2011).

23. Meyer, L. B. *Emotion and Meaning in Music*. (The Universtiy fo Chicago Press, 1956).

24. Xu, J., Wang, J., Fan, L., Li, H. & Zhang, W. Tractography-based Parcellation of the Human Middle Temporal Gyrus. *Sci. Rep.* 1–13 (2015). doi:10.1038/srep18883

25. Noonan, M. P., Kolling, N., Walton, M. E. & Rushworth, M. F. S. Re-evaluating the role of the orbitofrontal cortex in reward and reinforcement. *Eur. J. Neurosci.* **35,** 997–1010 (2012).

26. Wallis, J. D. Cross-species studies of orbitofrontal cortex and value-based decision-making. *Nat. Neurosci.* **15,** 13–9 (2012).

27. Kahnt, T., Heinzle, J., Park, S. Q. & Haynes, J.-D. The neural code of reward anticipation in human orbitofrontal cortex. *Proc. Natl. Acad. Sci. U. S. A.* **107,** 6010–5 (2010).
